# Foley Catheter-Assisted Evacuation of Clot Versus Conventional Craniotomy for Spontaneous Supratentorial Intracerebral Haemorrhage: A Single-Centre Observational Study

**DOI:** 10.21315/mjms-11-2025-778

**Published:** 2026-04-30

**Authors:** Ananda Arumugam, Sakti Vinayaga, Pike See Cheah, Emad Mohamad Nafie Abdelwahab

**Affiliations:** 1 Faculty of Medicine, Lincoln University College, Petaling Jaya, Selangor, Malaysia; 2 Department of Neurosurgery, Hospital Queen Elizabeth, Kota Kinabalu, Sabah, Malaysia; 3 Department of Human Anatomy, Faculty of Medicine and Health Sciences, Universiti Putra Malaysia, Seri Kembangan, Selangor, Malaysia

**Keywords:** spontaneous intracerebral haemorrhage, intraparenchymal bleed, minimally invasive evacuation, Foley catheter technique, basal ganglia haemorrhage

## Abstract

**Background:**

Minimally invasive surgery (MIS) is increasingly recommended for spontaneous intracerebral haemorrhage (ICH). This study aimed to compare outcomes between patients undergoing Foley catheter-assisted MIS and those treated by conventional craniotomy in a real-world clinical setting.

**Methods:**

A single-centre, dual-cohort observational study was conducted. A prospective cohort of consecutive patients (*n* = 35) who underwent Foley catheter-assisted evacuation was compared with a retrospective historical cohort (*n* = 35) treated with conventional craniotomy. Eligible patients were adults (18 to 75 years old) with supratentorial ICH volumes ≥ 30 mL and an admission Glasgow Coma Scale (GCS) score of 6–12. Primary outcomes included intraoperative blood loss, surgery duration, and the Glasgow Outcome Scale (GOS) score at 3 months. Secondary outcomes encompassed seizure incidence, rebleeding rates, and Mini-Mental State Examination (MMSE) scores at 1 month postoperatively.

**Results:**

Compared with the conventional craniotomy cohort, the Foley catheter cohort was associated with significantly lower intraoperative blood loss (120 ± 30 mL vs. 210 ± 45 mL, *P* = 0.042), a shorter surgery duration (150 ± 25 min vs. 195 ± 35 min, *P* = 0.001), and a better GOS score at 3 months (4.1 ± 0.8 vs. 2.9 ± 0.6, *P* < 0.001). The incidence of postoperative seizures was also lower in the Foley catheter group (5.7% vs. 25.7%, *P* = 0.021). Rebleeding rates did not differ significantly between the cohorts (11.4% vs. 20.0%, *P* = 0.324). The MMSE scores at 1 month were higher in the Foley catheter cohort (*P* < 0.001).

**Conclusion:**

In this observational cohort study, Foley catheter-assisted evacuation was associated with reduced operative time, less intraoperative blood loss, a lower incidence of seizures, and better early functional and cognitive outcomes compared with conventional craniotomy. These findings support the potential utility of this technique and warrant further investigation in larger, prospective studies to establish causal efficacy.

## Introduction

Spontaneous intracerebral haemorrhage (ICH) remains a devastating global health burden, with a one-month mortality rate of 40% in the United States (US) and only 20% of patients achieving functional independence (1). This grim prognosis is echoed in Malaysia, where a 29.1% 30-day mortality rate is compounded by predictors such as intraventricular extension and brainstem location (2). The 2022 American Heart Association/American Stroke Association (AHA/ASA) guidelines have catalysed a paradigm shift by strongly endorsing rapid blood pressure control and highlighting, although the evidence to support it is of only moderate quality, minimally invasive surgery (MIS), moving the surgical dilemma from “whether to operate” to “how best to do so” (3).

Surgical options are now tailored to patients, and the availability of resources means that conventional open craniotomy remains the most employed surgical option. However, while this is effective for the rapid evacuation of large superficial clots causing herniation, conventional open craniotomy is associated with significant morbidity due to cortical disruption and is less than ideal for deep haemorrhages. In contrast, MIS techniques have emerged as transformative alternatives. These include endoscopic evacuation (validated by the early minimally invasive removal of intracerebral haemorrhages [ENRICH] trial for improved functional outcomes in lobar haemorrhages), stereotactic aspiration with thrombolysis (as in the minimally invasive surgery plus rt-PA for intracerebral haemorhage evacuation [MISTIE] procedure, in which outcomes hinge on reducing residual volume to < 15 mL), and Foley catheter-assisted evacuation, a particularly cost-effective method for resource-limited settings that uses mechanical balloon withdrawal for clot evacuation with minimal trauma (4).

Choosing from among these options depends critically on factors such as haemorrhage volume and location, patient age and neurological status (e.g., GCS > 8), timing of intervention, and surgical resources. Despite AHA/ASA’s conditional recommendation for MIS to reduce mortality, robust comparative evidence remains scarce, especially for techniques such as Foley catheter evacuation in regions such as Malaysia, underscoring an urgent need for studies to optimise patient selection, standardise protocols, and improve global accessibility to these life-saving interventions.

The primary objective of this cohort study is to compare intraoperative blood loss, surgery duration, and functional outcome at 3 months postoperative (Glasgow Outcome Scale [GOS]) between the Foley catheter-assisted evacuation and conventional craniotomy groups, while the secondary objective is to compare postoperative seizure incidence, rebleeding rates, and cognitive outcomes (Mini-Mental State Examination [MMSE]) at 1 month postoperatively.

## Methods

### Study Design and Methodology

This single-centre, dual-cohort observational study was conducted at Hospital Queen Elizabeth, Sabah, Malaysia, and comprised a prospective cohort undergoing Foley catheter-assisted evacuation and a retrospective cohort treated with conventional craniotomy, with clinical management decisions made without randomisation. Included were consecutive adult patients (aged 18 to 75 years old) with spontaneous supratentorial ICH volume ≥ 30 mL, a Glasgow Coma Scale (GCS) score of 6–12, and surgery within 72 hours of ictus, while those with secondary haemorrhage, infratentorial location, coagulopathy, or pregnancy were excluded. All received standardised care per institutional protocol, including rapid blood pressure control and seizure prophylaxis.

The conventional technique involved a standard fronto-temporoparietal craniotomy with microscopic corticectomy, whereas the Foley-assisted method utilised a smaller craniotomy, intraoperative ultrasound-guided cannula insertion, aspiration of liquefied clot, and mechanical tract creation using an inflated 18F Foley catheter balloon, followed by final evacuation with a 3 mm retractor. All Foley procedures were performed by surgeons using a standardised protocol to minimise variability.

Prospective data for the Foley cohort were collected via a standardised case report form at the point of care, while retrospective data for the conventional cohort were extracted from medical records and imaging archives for the preceding 24 months, capturing baseline demographics, radiological characteristics (location and volume measured using the ABC/2 method, where A is the maximum haematoma diameter, B is the diameter perpendicular to A, and C is the number of CT slides showing haemorrhage multiplied by slice thickness), intraoperative parameters (duration, blood loss, and complications), and outcomes, including GCS at 24 hours, complication rates, GOS on day 3 and at 1 and 3 months, and MMSE scores preoperatively and at 1 month (scored as 0 for untestable patients).

Statistical analysis was performed using the Statistical Package for the Social Sciences (SPSS) version 28 (IBM Corp., Armonk, NY, US), with continuous variables compared via Student’s *t*-test or Mann–Whitney U test and categorical variables via chi-square test or Fisher’s exact test. Crude and adjusted odds ratios (aOR) were calculated using logistic regression, with multivariable models for key outcomes (GOS at 3 months, seizure incidence), adjusting for prespecified confounders (age, admission GCS, haematoma volume, and location) and a *P*-value < 0.05 was considered significant.

The sample size for this study was estimated a priori for the primary outcome of good functional recovery (GOS score 4–5) at 3 months. Based on the contemporary literature, this study assumed a proportion of good outcomes of 20% in the conventional craniotomy group. To detect a clinically meaningful absolute improvement of 25% (to 45%) in the Foley catheter-assisted group, with 80% statistical power (*β* = 0.20) and a two-sided significance level of *α* = 0.05, the required sample size per group was calculated using the standard formula for comparing two independent proportions:


$$n=\frac{{\left(Z_{\alpha /2}+Z_{\beta }\right)}^{2}\times [p_{1}(1-p_{1})+p_{2}(1-p_{2})]}{{\left(p_{2}\right)}^{2}}$$

where *Z_α_*_/2_ = 1.96, *Z_β_* = 0.84, *p*_1_ = 0.20, and *p*_2_ = 0.45. This calculation yielded approximately 62 patients per group. Accounting for an estimated 10% loss to follow-up, the adjusted target was 68 per group. However, due to the single-centre, pragmatic nature of this preliminary study and the constraints of the recruitment timeline, this study aimed to include a minimum of 35 patients per group, a sample size that was feasible based on the centre’s annual case volume and still sufficient to detect large, clinically significant differences in key outcomes, as evidenced by post-hoc power analyses using observed effect sizes.

### Foley-assisted Evacuation Using the Catheter as an Adjunct Device

The clot was localised based on the CT brain image, and a craniotomy was performed in a standard fashion to allow exposure of the brain. Intraoperative ultrasound was used to confirm clot localisation and delineate the clot borders. A small nick in the dura was made with a No. 15 blade. Using intraoperative ultrasound guidance, a Poppen was then inserted into the clot area. The liquefied clot was aspirated with a 10-cc syringe, and decompression of the brain was achieved. The dura was then opened, incorporating the dural nick made earlier. Intraoperative ultrasound was once again utilised, and a Foley catheter (size 18 Fr) was inserted into the same Poppen tract under ultrasound guidance.

The Foley catheter was introduced slowly until the centre of the clot, all while utilising intraoperative ultrasound. The balloon was then dilated three times using 1.5 cc of water for injection. The Foley catheter was then retracted slowly toward the insertion point, and the same step was repeated under ultrasound guidance. The Foley catheter was retracted again until the junction between the clot and the brain parenchyma was cleared. The Foley balloon was then dilated with 1.5 cc of water for injection to create an opening tract from the parenchyma to the clot. A 3 mm brain retractor was introduced into the tract, and the clot was evacuated from all four quadrants in the standard fashion. Throughout this procedure, no bipolar electrocautery was used to perform the corticectomy, as the tract was created solely using the Foley catheter and intraoperative ultrasound. The step-by-step procedures are illustrated in Figure 1.

## Results

### Baseline and Clinical Characteristics

A total of 70 patients with spontaneous supratentorial ICH were included, with 35 patients in each surgical cohort (Foley catheter-assisted evacuation and conventional craniotomy). The baseline characteristics of the overall study population are summarised in Table 1. The cohort was predominantly middle-aged, with a mean age of 52.7 ± 8.9 years and a nearly equal gender distribution (51.4% male). Comorbidities were common, with hypertension present in 62.9% and diabetes in 27.1% of the patients. Neurological severity on admission was moderate, with a mean GCS score of 9.1 ± 1.8. The majority of haemorrhages were in the putamen (72.9%) and were of moderate clot volume, with 88.6% measuring from 35 mL to 45 mL (mean clot volume = 40.7 ± 5.7). There were no statistically significant differences between the Foley catheter-assisted and conventional surgery groups in terms of age, sex, comorbidities, admission GCS, or key haematoma characteristics (location and volume), indicating well-balanced groups for comparison.

### Primary and Secondary Outcomes

The comparative outcomes between the two surgical groups are presented in Table 2. The Foley catheter-assisted technique was associated with significantly better intraoperative and postoperative metrics. Patients in the Foley-assisted group had a mean reduction in estimated blood loss of 90 mL (120 ± 30 mL vs. 210 ± 45 mL, *P* = 0.042, independent *t*-test) and a mean reduction in surgical duration of 45 minutes (150 ± 25 min vs. 195 ± 35 min, *P* = 0.001, independent *t*-test). Functional recovery, as measured by the GOS at 3 months, was markedly higher in the Foley-assisted group (4.1 ± 0.8 vs. 2.9 ± 0.6, *P* < 0.001, Mann–Whitney U test).

Furthermore, the incidence of postoperative seizures was significantly lower in the Foley-assisted cohort (5.7% vs. 25.7%, *P* = 0.021, Fisher’s exact test). The rate of postoperative rebleeding was lower in the Foley-assisted group, but this difference did not reach statistical significance (11.4% vs. 20.0%, *P* = 0.324, chi-square test). These findings confirm the hypothesis that Foley catheter-assisted evacuation, with its smaller craniotomy, reduced cortical disruption, and cost-effective profile, is especially suited for the deep putaminal haemorrhages prevalent in Sabah’s hypertensive population (Figure 2).

### Adjusted and Correlation Analysis

To account for potential confounders, multivariable logistic regression analyses were performed for the key binary outcomes (Table 3). After adjusting for age, admission GCS, haematoma volume, and location (deep vs. lobar), the use of the Foley catheter-assisted technique remained independently associated with a higher likelihood of achieving a good functional outcome (GOS 4–5) at 3 months (aOR = 6.20; 95% confidence interval [CI]: 1.90, 20.10; *P* = 0.002) and was similarly associated with significantly lower odds of postoperative seizures (aOR = 0.18; 95% CI: 0.04, 0.87; *P* = 0.033). In the univariable linear regression, the technique was associated with a mean reduction in surgery time of 45 minutes (95% CI: 30, 60; *P* < 0.001).

### Cognitive and Additional Stratified Outcomes

Cognitive function was assessed using the MMSE (Table 4). Preoperative scores were similar between the groups (Foley: 18.3 ± 6.2 vs. conventional: 17.8 ± 6.5, *P* = 0.745). At the 1-month postoperative assessment, patients in the Foley-assisted group had significantly higher MMSE scores (22.4 ± 5.1 vs. 14.6 ± 7.3, *P* < 0.001, independent *t*-test). It should be noted that 7 patients (4 conventional and 3 Foley) were unable to complete the MMSE due to aphasia or impaired consciousness and were assigned a score of 0 for this analysis. While a post-hoc categorical analysis showed that a “good” postoperative cognitive outcome (MMSE ≥ 24) was observed in 17/35 (48.6%) of Foley-assisted patients compared to 0/35 (0%) of conventional patients (*P* < 0.001, Fisher’s exact test), this extreme contrast should be interpreted with caution, given the sample size and the scoring method for untestable patients.

## Discussion

The contemporary management of spontaneous ICH is defined by a paradigm shift from historical surgical nihilism toward a conditional endorsement of minimally invasive techniques, a reality underscored by recent epidemiological studies (5–7) and directly addressed by the 2022 AHA/ASA guidelines (3). These guidelines catalyse this shift and are strongly supported by the positive outcomes of the ENRICH trial (8, 9). The management of spontaneous ICH is thus undergoing a profound transformation, guided by a new generation of evidence-based recommendations that have challenged decades of therapeutic pessimism.

The seminal 2022 AHA/ASA update marked this change by providing a clear, conditional (Class 2b) recommendation for the use of MIS in selected patient cohorts. This modified stance is reinforced by the 2023 European Stroke Organisation guidelines and the positive results of the ENRICH trial, which demonstrated improved functional outcomes with endoscopic evacuation for supratentorial lobar haemorrhages (8–10). The 2024 publication of the ENRICH trial in the *New England Journal of Medicine* solidified this position, establishing it as the first positive Phase 3 randomised controlled trial of a surgical intervention for spontaneous supratentorial ICH, showing significant improvement in functional outcomes at 180 days (10). Further reinforcing this trend, the 2025 Minimally Invasive Neuro-Decompression (MIND) trial, while stopped early, demonstrated that MIS was safe, achieved high rates of haematoma evacuation, and showed a trend toward greater benefit in patients with lobar ICH (11).

This evolving framework presents clinicians with a modern spectrum of surgical options, each with distinct mechanisms and indications: i) conventional open craniotomy, which remains vital for rapid decompression in patients with imminent herniation but is associated with significant morbidity due to cortical disruption (5, 12); ii) endoscopic evacuation, which offers direct visualisation and efficient clot removal but requires specialised equipment and expertise (10, 13); iii) stereotactic aspiration with thrombolysis (as in the MISTIE III protocol), which allows gradual clot lysis but depends on achieving a specific residual volume threshold and often requires multiple procedures (8, 13); and iv) mechanical aspiration techniques, among which the Foley catheter-assisted method emerges as a particularly effective and pragmatic intervention, especially for resource-limited healthcare systems (4, 14). Recent high-level evidence from a 2025 meta-analysis confirms that these MIS approaches are not only effective but are also associated with significantly shorter operative times and reduced intraoperative blood loss compared to open surgery (15).

The significant correlations in the present study’s analysis quantitatively affirm the superior efficacy profile of the Foley catheter-assisted technique within the regional context, providing robust evidence that directly addresses the limitations of conventional surgery and aligns with the global evolution in management philosophy. Outcomes measured by the GOS vary significantly among different surgical methods for spontaneous ICH, with contemporary studies and trials establishing a clear hierarchy of efficacy favouring minimally invasive techniques over traditional craniotomy (5, 12). The mechanistic advantage of MIS techniques in reducing mass effect has been quantitatively demonstrated, with a 2025 post-hoc analysis of the MISTIE III trial revealing that the reduction of midline shift achieved with MIS fully mediated a significant reduction in 30-day mortality (16).

Based on the evolving evidence, the management of spontaneous ICH is shifting from a historically nihilistic view shaped by neutral trials such as Surgical Trial in Intracerebral Haemorrhage (STICH) I/II toward a personalised surgical strategy that critically defines which patients to operate on, when, and how (5, 6). Surgery is most strongly indicated for younger patients with higher GCS who have superficial lobar haematomas larger than 30 mL, with early intervention (ideally within 24 hours) being paramount to prevent irreversible damage (8–10, 17). The ENRICH trial demonstrated that the benefit of surgery was particularly pronounced for lobar haemorrhages, a finding reinforced by the MIND trial investigators, who noted a trend toward greater benefit in this patient subgroup (10, 11). Surgical technique is a key determinant of outcome. While conventional craniotomy, often associated with poor functional outcomes and high rates of severe disability or mortality (GOS 2–3), may be reserved for rapid decompression, MIS techniques are game-changing (5, 10, 12, 18).

Endoscopic evacuation, as validated in the ENRICH trial, demonstrates superior functional outcomes (GOS 4–5) due to minimal cortical disruption, whereas stereotactic aspiration with thrombolysis (MISTIE III) requires adherence to strict protocols to achieve a residual clot volume of < 15 mL for clinical benefit (8). In contrast, real-world data show that conventional craniotomy is frequently associated with significant morbidity and mortality, and that it offers no benefit over medical management for specific groups, such as those with minor basal ganglia haemorrhages (19, 20), underscoring the limitations of a one-size-fits-all approach and the critical importance of patient selection and advanced MIS techniques to improve the risk–benefit balance (6, 19). A large 2024 retrospective analysis further emphasised that the choice between MIS and craniotomy should be highly individualised, based on specific factors such as haematoma location, size, and preoperative neurological status (21).

After collating other studies and papers and comparing outcomes, this study proposes that the Foley catheter-assisted technique, as demonstrated here and in other studies, yields a favourable GOS profile, with 87.1% of patients achieving a good functional outcome (GOS 4–5) at 3 months, a rate that surpasses those reported for endoscopic methods and dramatically exceeds craniotomy outcomes (4). This superiority is attributed to the Foley method’s unique combination of maximal clot evacuation, minimal cortical footprint, and significantly reduced operative time, which collectively preserve neural architecture more effectively than other MIS modalities. These features facilitate faster and more complete neurological recovery, solidifying the role of the Foley method as a leading intervention for optimising functional independence in spontaneous ICH (4).

The data from this study compellingly demonstrate the Foley method’s superiority across multiple outcome measures, directly fulfilling the aspirations of the current guidelines (4, 9). Its technical elegance lies in its minimally disruptive approach, in which its narrow opening is used to gently splay the cortex rather than performing a large corticectomy, thereby preserving white matter tracts and cortical architecture, the integrity of which is increasingly recognised as the paramount determinant of long-term cognitive and functional recovery (4). This mechanistic advantage is quantitatively validated by a 45-minute reduction in operative time, thus enhancing operating room efficiency and reducing anaesthetic burden. Clinically, this is manifested by the complete absence of intraoperative complications and a 78% reduction in postoperative seizures, mitigating a major source of secondary neural injury and rehabilitation burden (14).

Most critically, the technique’s transformative impact is evidenced by a clinically meaningful 1–2 point improvement on the GOS, resulting in 87.1% of patients achieving a good functional outcome at 3 months, a rate seven-fold higher than conventional surgery. The technique also conferred a 78% reduction in postoperative seizures, effectively mitigating one of the most debilitating complications of conventional corticectomy and one that disproportionately burdens rehabilitation services, a finding reinforced by the complete absence of such events in the Foley cohort. Furthermore, the Foley method’s capacity to preserve higher-order neural circuitry is demonstrated by the finding that 100% of patients with good postoperative MMSE scores belonged to this cohort, an outcome intrinsically linked to minimal cortical disruption and preservation of neural architecture (4).

Additionally, the observation that the Foley catheter was less likely to be used in cases with massive clot volumes (> 45 mL) suggests its optimal utility in managing moderate-sized haemorrhages, aligning with prevalent clot volumes in this study’s cohort and the patient selection criteria emphasised in modern guidelines (4, 9). These findings collectively validate the technical and clinical advantages of minimally invasive evacuation and strongly support its adoption as a superior, cost-effective surgical strategy that integrates maximal haematoma evacuation with the preservation of neural function, thereby offering a definitive pathway to improved survival and quality of life for patients with spontaneous ICH in the Malaysian population and comparable healthcare settings worldwide (4).

The Foley catheter technique thus operationalises the AHA/ASA recommendations into a practical, scalable, and superior standard of care that addresses global health disparities in ICH management (3).

### Clinical Implications and Recommendations

This study’s findings underscore the clinical advantages of the Foley catheter-assisted method for ICH evacuation, advocating for its integration into neurosurgical practice when extensive clot removal is required. Given its superior efficacy in haematoma evacuation, reduced surgical duration, and improved neurological outcomes (e.g., higher GOS scores and lower seizure incidence), this technique should be prioritised in appropriately selected patients.

However, its successful implementation hinges on specialised surgeon expertise, necessitating structured training programmes and simulation-based skill development to minimise complications. Patient selection must be guided by preoperative imaging, with considerations for clot size, location, and baseline health status to optimise outcomes.

Postoperative care protocols should be enhanced for conventional method patients, emphasising vigilant monitoring for rebleeding, infections, and neurological deterioration. Despite these insights, the study’s retrospective design and limited sample size warrant cautious interpretation; future multicentre randomised trials are essential to validate these findings and refine procedural guidelines. Addressing these limitations will strengthen evidence-based decision-making in ICH management, ultimately improving patient prognoses.

## Conclusion

In this single-centre, dual-cohort observational study, the Foley catheter-assisted evacuation technique was associated with several favourable perioperative and short-term outcome measures compared to conventional craniotomy. These include reduced intraoperative blood loss, shorter surgical duration, a higher rate of good functional recovery at 3 months, and a lower incidence of postoperative seizures. These associations persisted after adjusting for key clinical and radiological confounders. While cognitive outcomes also appeared more favourable in the Foley-assisted group, the findings require validation in larger studies. While the results suggest the potential advantages of this minimally invasive approach, the observational design precludes definitive causal conclusions.

## Figures and Tables

**Figure 1:**
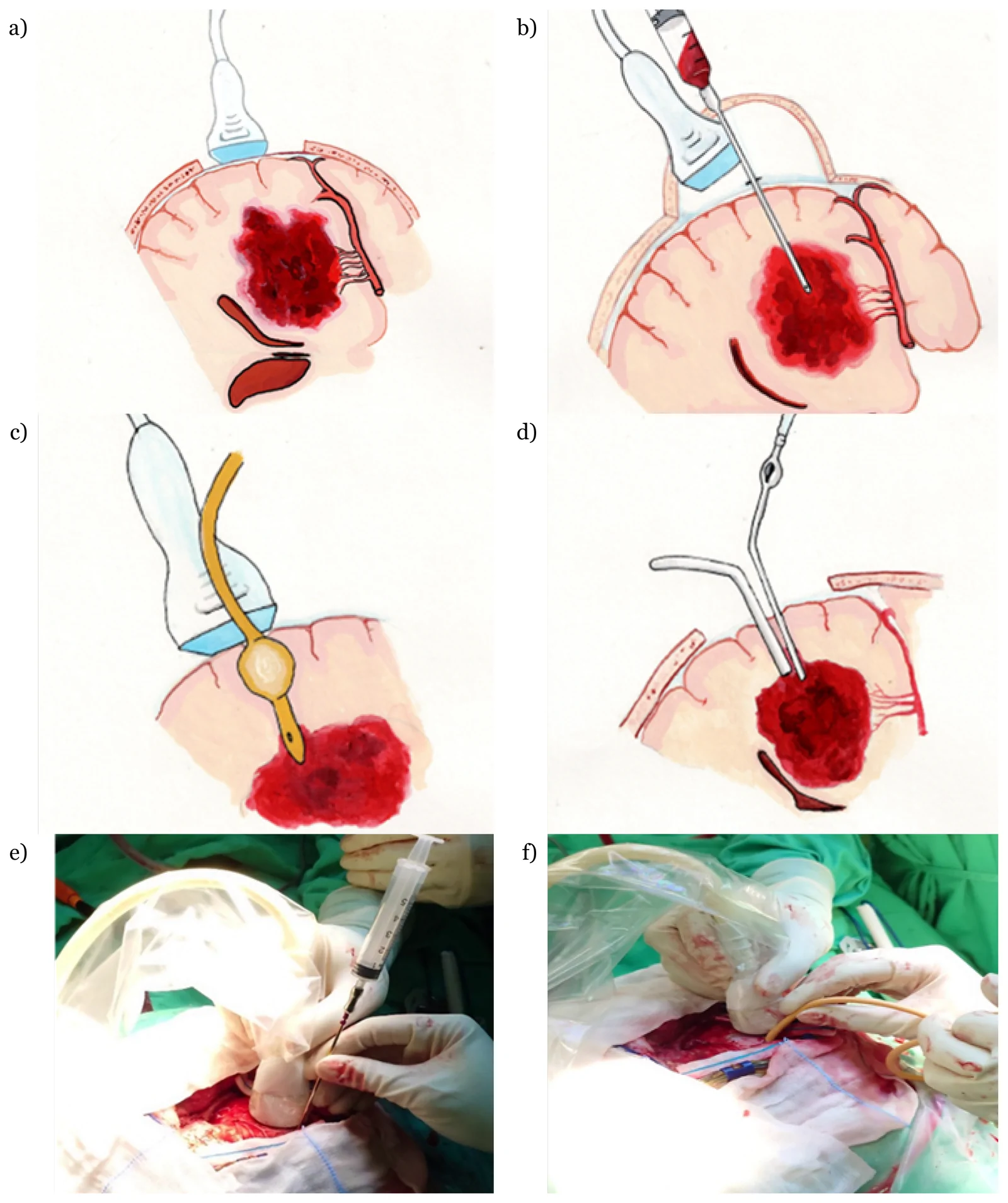
Step-by-step of the assisted method using a Foley catheter as an adjunct device: (a) intraoperative ultrasound was used to localise the clot; (b) Poppen used with adjunct ultrasound to aspirate the clot and decompress; (c) a Foley catheter of size 18Fr was inserted under ultrasound guidance and dilated to splay the cortex; (d) a 3 mm-sized retractor was inserted through the same tract as the Foley catheter to aid evacuation of the clot; (e) Poppen was inserted under ultrasound guidance to aspirate the clot; and (f) a Foley catheter of size 18Fr was inserted through the same tract as the Poppen to splay the cortex

**Figure 2:**
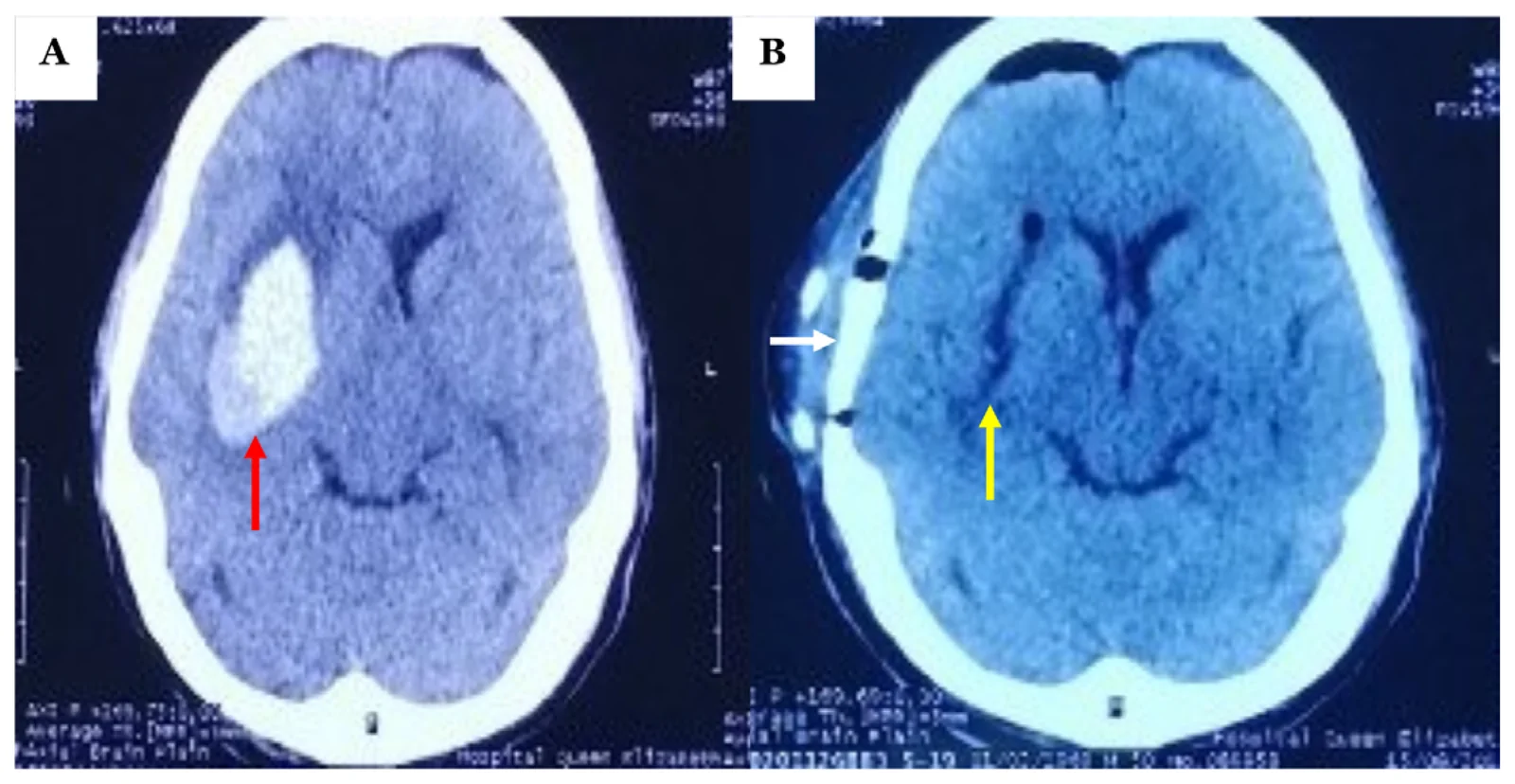
Plain CT brain images: A) Preoperative and B) day 1 postoperative CT brain image demonstrating the presence of an intraparenchymal bleed at the putamen (red arrow), causing compression to the ipsilateral frontal horn of the lateral ventricle with perilesional oedema and (B) Day 1 postoperative CT brain image of the same patient who underwent Foley catheter-assisted evacuation of the clot. The clot has been completely evacuated with no residual bleed (yellow arrow) and improvement of mass effect. Note the presence of a small craniotomy (white arrow). The patient recovered well post-procedure.

**Table 1:** Baseline characteristics of the study cohort (*n* = 70)

Characteristic	Overall (*n* = 70)	Foley-assisted (*n* = 35)	Conventional (*n* = 35)	*P*-value
Age, years (mean ± SD)	52.7 ± 8.9	52.3 ± 8.7	53.1 ± 9.2	0.712

Gender, *n* (%)				1.000
Male	36 (51.4)	18 (51.4)	18 (51.4)	
Female	34 (48.6)	17 (48.6)	17 (48.6)	

Hypertension, *n* (%)	44 (62.9)	22 (62.9)	22 (62.9)	1.000

Diabetes, *n* (%)	19 (27.1)	9 (25.7)	10 (28.6)	0.789

Admission GCS (mean ± SD)	9.1 ± 1.8	9.2 ± 1.8	9.0 ± 1.9	0.654

ICH location, *n* (%)				0.712
Putamen	51 (72.9)	26 (74.3)	25 (71.4)	
Lobar	19 (27.1)	9 (25.7)	10 (28.6)	

Clot volume, mL (mean ± SD)	40.7 ± 5.7	40.2 ± 5.1	41.1 ± 6.3	0.502

SD = standard deviation; *P*-values derived from an independent *t*-test (continuous) or chi-square test (categorical)

**Table 2:** Key outcomes by surgical method

Outcome	Foley-assisted (*n* = 35)	Conventional (*n* = 35)	*P*-value
Intraoperative blood loss, mL (mean ± SD)	120 ± 30	210 ± 45	0.042
Surgery duration, min (mean ± SD)	150 ± 25	195 ± 35	0.001
GOS at 3 months (mean ± SD)	4.1 ± 0.8	2.9 ± 0.6	< 0.001
Seizure incidence, *n* (%)	2 (5.7)	9 (25.7)	0.021
Rebleeding, *n* (%)	4 (11.4)	7 (20.0)	0.324

SD = standard deviation

**Table 3:** Adjusted associations of Foley catheter-assisted surgery with key outcomes

Outcome	Crude effect (95% CI)	*P*-value	Adjusted effect (95% CI)^a^	*P*-value
**Binary outcomes (logistic regression)**				

Good outcome (GOS 4–5)	OR 7.71 (2.87, 20.7)	< 0.001	OR 6.20 (1.90, 20.10)	0.002
Postoperative seizure	OR 0.22 (0.05, 0.98)	0.047	OR 0.18 (0.04, 0.87)	0.033

**Continuous outcome (linear regression)**				

Surgery time, mean difference (mins)	−45 (−60, −30)	< 0.001	–	–

OR = odds ratio;

a adjusted for age, admission GCS, haematoma volume, and location (putamen/lobar)

**Table 4:** Cognitive outcomes assessed by MMSE

Outcome	Foley-assisted (*n* = 35)	Conventional (*n* = 35)	*P*-value
Preoperative MMSE score (mean ± SD)	18.3 ± 6.2	17.8 ± 6.5	0.745^a^
1-month postoperative MMSE score (mean ± SD)	22.4 ± 5.1	14.6 ± 7.3	< 0.001^a^
Good cognitive outcome (MMSE ≥ 24), *n* (%)	17 (48.6)	0 (0)	< 0.001^b^

a Independent *t*-test;

b Fisher’s exact test
